# Biogeographic variation in mussel shell thickness and drilling predation on rocky shores

**DOI:** 10.1007/s00442-025-05760-x

**Published:** 2025-07-08

**Authors:** Emily K. Longman, Eric Sanford

**Affiliations:** 1 Bodega Marine Laboratory, University of California Davis, Bodega Bay, CA 94923 USA; 2 Department of Evolution and Ecology, University of California Davis, Davis, CA 95616 USA; 3 Present Address: Department of Biology, University of Vermont, Burlington, VT 05405 USA

**Keywords:** Adaptive landscape, Local adaptation, *Mytilus californianus*, *Nucella canaliculata*, Predator–prey interaction, Selection mosaics

## Abstract

Although predator and prey species often interact over broad geographic ranges, little consideration has been given to whether environmentally-driven variation in prey traits might create a landscape of selection that shapes the evolution of predator traits. Here, we investigate whether predatory intertidal snails (the dogwhelk *Nucella canaliculata*) vary in their drilling capacity in association with prey defenses (mussel shell thickness) over ~ 1000 km of coastline in the northeast Pacific. We analyzed mussels (*Mytilus californianus*) collected over two decades and found that shells from central Oregon were historically ~ 20% thicker than those from California. We tested the drilling capacity of dogwhelks raised under common-garden laboratory conditions and found that dogwhelks from California could drill mussels 3.4 times thicker than those from Oregon. Thus, dogwhelks with stronger drilling ability are associated with thinner mussel shells. Although these findings appear counterintuitive at first, they are consistent with strong prey-driven selection on the predator in California, and a region of weak selection in Oregon where mussels may be too thick to favor drilling. Our results also suggest that this selective landscape may be changing; consistent with recent studies of ocean acidification, mussels collected in 2019 were thinner than in prior decades, particularly in Oregon. Overall, our study highlights the importance of studying species interactions within an explicit geographic context of shifting selection pressures.

**Supplementary Information:**

The online version contains supplementary material available at 10.1007/s00442-025-05760-x.

## Introduction

Understanding the processes that generate and maintain phenotypic variation across a landscape is a fundamental challenge that lies at the intersection of ecology and evolution. Landscapes are composed of mosaics of abiotic and biotic factors that can impose spatially varying selection on populations. If these selective forces are consistent over time, species may adapt to their surrounding environments, such that local populations perform better than populations from farther away (Kawecki and Ebert 2004; Blanquart et al. 2013). These patterns of local adaptation can influence ecological dynamics ranging from changes in population abundance to community diversity to ecosystem properties (Urban et al. 2020). Patterns of evolutionary divergence and the associated ecological effects are the basis for most eco-evolutionary dynamics (Hendry 2017).

Predator–prey species interactions are ideal systems for studying eco-evolutionary dynamics, because the risk of mortality from predation or from insufficient food consumption can create strong reciprocal selection between two interacting species, often leading to an evolutionary arms race (Vermeij 1994; Abrams 2000; Brodie et al. 2002; Thompson 2002). Landscapes are heterogeneous, thus studying predator–prey interactions across a range of environments is important for teasing apart patterns of selection and coevolution (Hand et al. 2015). The geographic mosaic theory of coevolution is a framework to study reciprocal selection in coevolving species across landscapes (Thompson 1999, 2002). Across the ranges of the two interacting species, there can be regions where selection imposed on one or both species is strong (‘hot spots’) and regions where selection is weak (‘cold spots’). The most extreme cold spots can occur if the geographic distributions of the two interacting species are not completely aligned, so that one species is absent from some regions (Brodie et al. 2002; Freeman and Byers 2006). Although less studied, selection cold spots can also occur when both species are present, but additional biotic (Benkman et al. 2001) or abiotic factors (Hochberg and Baalen 1998) alter the strength of the interaction between the two species. More specifically, if the morphological or physiological traits of one species vary geographically due to environmental forces, this may create a unique selective landscape for the interacting prey or predator (Toju 2008). In addition, species life history, geographic barriers to dispersal, and population structure can add further complexity to the mosaic of coevolution across a landscape (Thorpe et al. 1995; Hague et al. 2020).

Most research on the coevolutionary dynamics of predator–prey interactions has focused on how predation pressure shapes the evolution of prey traits (McLean et al. 2024; Urban et al. 2020). This bias is due to the assumed asymmetry in coevolving species, with stronger selection on prey defenses than predator attack strategies, because prey are fleeing for their lives, while predators are only running for their dinner (Dawkins and Krebs 1979). However, many factors (e.g., whether prey are toxic or highly valued in a predator’s diet) can skew coevolutionary feedbacks, such that there is instead stronger selection on predators than prey (Humphreys and Ruxton 2020; McLean et al. 2024). In addition, prey abundance (Turner 2005), nutritional value (Daugherty 2011), and defenses (Hahn et al. 2019) can vary spatially due to environmental factors and can alter the strength of selection on predators. For example, latitudinal gradients in temperature can influence growth and calcification in marine molluscs, which may influence their vulnerability to predation (Mancuso et al. 2019). Evolved differences in predator traits can in turn drive strong eco-evolutionary effects in the surrounding community through both direct and indirect effects on other species (Schmitz 2017). However, to date, there have been few empirical studies of how latitudinal variation in prey traits might impose selection on the evolution of predator traits (but see Brodie et al. 2002; Kraaijeveld and Godfray 1999; Hague et al. 2020).

Understanding the adaptive landscape of selection that shapes species interactions also requires examining temporal variation in geographic gradients or mosaics of abiotic conditions. This landscape may shift continually through time with biotic and environmental changes (Burak et al. 2018), including unprecedented rates of recent climate change that can differentially affect interacting species (Tylianakis et al. 2008). These changes across a landscape may shift selection hot spots to cold spots, or vice versa (Duncan et al. 2017). Changes could arise via range shifts or contractions, such that predator and prey may no longer interact in some regions, or a third species may shift its range and influence the dynamic of the focal predator and prey species (Parmesan and Yohe 2003; Chen et al. 2011). Alternatively, abiotic changes may alter the traits of a given species, which could subsequently modify how two species interact (De Block et al. 2013; Harvey and Moore 2017; Kroeker and Sanford 2022). Having a solid understanding of current and past selective landscapes is thus an important foundation for understanding eco-evolutionary dynamics of interacting species as environments change. However, there have been few empirical studies of whether or not the geographic gradients or mosaics of environmental conditions that shape interactions between two species are consistent through time. Therefore, in this study, we investigated whether spatial and temporal variation in prey traits might shape a predator–prey interaction over ~ 1000 km of coastline.

The Channeled Dogwhelk, *Nucella canaliculata,* is a predatory snail (family Muricidae) found in rocky intertidal ecosystems ranging from central California to Alaska. One of its primary prey is the mussel *Mytilus californianus*, a critical foundation species that creates large mussel beds along the west coast of North America (Sanford et al. 2003; Sanford and Worth 2009, 2010). Muricid dogwhelks consume prey by a chemo-mechanical mode of feeding in which they use acid secretions and their radula to drill a tiny hole through the shell of their prey (Carriker 1981).

The interaction between *N. canaliculata* and the mussel *Mytilus californianus* provides a promising study system to address how spatial and temporal variation in prey traits might shape the evolution of predator traits. Previous research has documented geographic variation in this predator–prey interaction (Sanford et al. 2003; Sanford and Worth 2009, 2010). In particular, dogwhelks from California readily drilled mid-sized (50–70 mm) mussels, while those from Oregon generally could not. This pattern was consistent through two generations and appears to have a genetic basis (Sanford and Worth 2009). Here, we hypothesize that mussel shell thickness varies along the West Coast and thus may influence selection on predator drilling capacity. The California Current along the Pacific Coast of North American is characterized by a persistent spatial mosaic of coastal upwelling, with associated variation in seawater temperature, pH, and chlorophyll-*a* (Kroeker et al. 2016; Chan et al. 2017). Variation in these oceanographic conditions can in turn influence patterns of mussel growth and shell thickness (Kroeker et al. 2016; Rose et al. 2020). If this spatial pattern of upwelling and oceanographic conditions has been persistent over long timescales, this may create a spatial mosaic of mussel shell traits along the West Coast with effects on the evolution of drilling capacity in *N. canaliculata*. For example, if there is strong reciprocal selection between predator and prey, regions along the coast with thick mussels may be associated with populations of dogwhelks with deep drilling phenotypes. Alternatively, if the selection landscape is primarily shaped by environmentally driven variation in prey defenses (i.e., mussel shell thickness), then dogwhelk drilling capacity may show a more complex pattern.

Restricted connectivity among populations of *N. canaliculata* increases the likelihood that this predator is adapted to a spatial mosaic of mussel shell thickness along the coast. *N. canaliculata* lay clusters of benthic egg capsules during the spring and summer. Young dogwhelks develop within the egg capsules, then hatch and crawl away, establishing in the surrounding area. *N. canaliculata* live within mussel beds on wave-exposed rocky headlands, so populations are separated along the coast by intervening sandy beaches. Consistent with their life history and habitat specificity, sequence data confirm that there is limited gene flow and connectivity among populations along the coast (Sanford et al. 2003).

In this study, we quantified variation in the drilling capacity of *N. canaliculata* on mussels along the coasts of California and Oregon. We raised dogwhelks from six populations from hatching to adult size in a common laboratory environment. We then assessed drilling capacity by quantifying the maximum length and maximum thickness of mussel shells that dogwhelks could drill through. To explore possible selective forces underlying this species interaction, we analyzed the thickness of archived and contemporary mussel shells spanning approximately two decades to determine if there has been a consistent or variable mosaic of selective pressures across space and time. Finally, we tested if variation in drilling ability among dogwhelk populations was associated with this mosaic of mussel shell thickness.

## Methods

### Laboratory experiment to quantify drilling capacity

#### Laboratory rearing

**Fig. 1:**
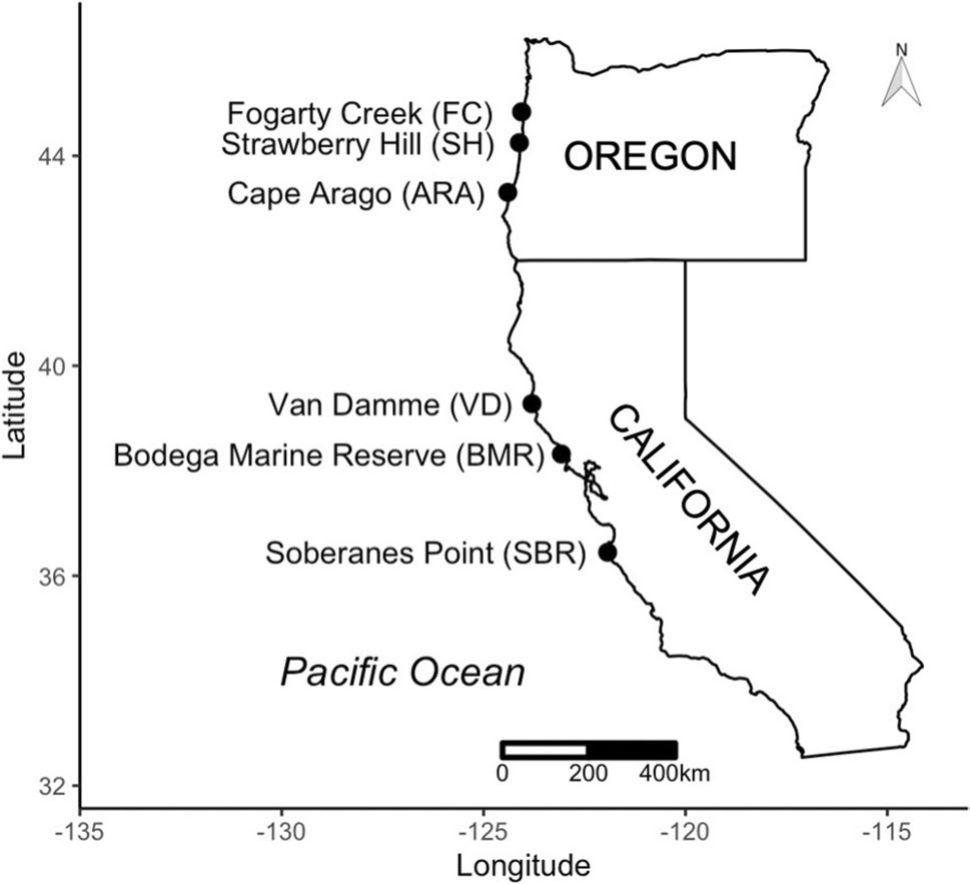
Map of study sites in California: Soberanes Point (36°26′50″N, 121°55′44″W), Bodega Marine Reserve (38°19′09″N, 123°04′28″W), Van Damme State Park (39°16′43″N, 123°48′12″W); and Oregon: Cape Arago (43°18′13″N, 124°24′07″W), Strawberry Hill (44°15′00″N, 124°06′55″W), and Fogarty Creek (44°50′16″N, 124°03′33″W)

*Nucella canaliculata* egg capsules were collected in May–June 2019 from six sites, three in California and three in Oregon (Fig. 1). At each site, 11–15 sets of egg capsules were collected; sets were separated by a minimum of 1 m to minimize the likelihood of relatedness. Egg capsules from a given set were held together in plastic tea strainers with mesh sides with ~ 180 μm openings (Upton Tea Imports, Holliston, Massachusetts, USA) and were assumed to contain dogwhelks that were full or half siblings, hereafter referred to as a “family.” Strainers were submerged in tanks of flowing seawater at Bodega Marine Laboratory. At the point of hatching, 80 juvenile dogwhelks from each family were transferred to a new tea strainer that was then placed in a 1-L plastic container on a sea table with flowing seawater. Dogwhelks were fed an ad libitum common diet of small blue mussel recruits, *Mytilus trossulus*, that were collected regularly from Bob Creek Wayside (44°14′39″N, 124°6′49″W) on the central Oregon coast. Containers were checked weekly to replenish food, remove any dogwhelks that died, and to clean containers. As dogwhelks grew, they received progressively larger *M. trossulus*. Once dogwhelks reached approximately 5 mm in length, they were transferred to a 1-L container with a partially mesh lid allowing for greater water flow and more space for growth.

### Laboratory scoring experiment

In June 2020, 10 dogwhelks from eight families from each of the six populations (*n* = 480 dogwhelks) were moved to individual 1-L containers with flow-through seawater. All dogwhelks started at an initial length of 18–22 mm. To standardize feeding history immediately prior to the start of the scoring experiment, each dogwhelk was fed 10 *M. trossulus* (10–20 mm long). In August 2020*, M. trossulus* were removed, the length of each dogwhelk was measured, and their sex was determined.

To determine variation in drilling capacity among dogwhelks, we quantified the maximum thickness of mussel shell that each dogwhelk could drill through during a 25-week experiment. To do so, we took advantage of the fact that larger *M. californianus* have thicker shells, and we challenged individual dogwhelks with a series of mussels of increasing size. Size classes (lengths) of mussels offered were: 20 mm (± 5 mm), 40 (± 5), 60 (± 10), 90 (± 10), 120 (± 10), 150 (± 10), and 170 (± 10). If a dogwhelk successfully drilled a mussel of one size class, it was subsequently offered thicker mussels from the next size class. This approach was taken because previous research indicated geographic variation in drilling ability (Sanford and Worth 2009) and a design that presented size classes in a random order would have resulted in a prohibitively long experiment that would likely have increased dogwhelk mortality. Instead, with the design we used, dogwhelks advanced to the mussel size/shell thickness that posed a challenge for their drilling capacity, and then had the remainder of the experiment to try to drill mussels of this size class. Mussels ranging from 20 to 150 mm were collected from Bodega Marine Reserve (BMR). Dogwhelks that progressed far enough in the series past the 150 mm mussel from BMR received a 150 mm mussel from Strawberry Hill (SH), and then a 170 mm mussel from SH. Based on preliminary data (Sanford, unpublished data), we assumed that the 150 and 170 mm mussels from Strawberry Hill would be exceptionally thick and represented the end point of our continuum. Using mussels from primarily one source population, we sought to control for unknown factors besides shell thickness (e.g., shell hardness and composition) that might vary among sites and influence our measurements of drilling capacity.

*N. canaliculata* containers were checked on a regular schedule (see Section S1) to quantify drilling. If a dogwhelk drilled a complete hole through a mussel from a given size class, it was then given a mussel(s) from the next size class. When a mussel was drilled or partially drilled, its length was measured. All drilled shells were kept for further analyses. After the completion of the experiment, the dogwhelks were measured to assess their growth.

### Shell thickness measurements in laboratory experiment

To assess how mussel shell thickness increased across the size classes, 20 mussels from each size class were cut using a bandsaw at 1/3 their length from the anterior end. This is the region of the shell that is most commonly drilled by *N. canaliculata* in the field (Longman and Sanford, personal observation). A random sample of drilled mussels from Soberanes Point, Bodega Marine Reserve and Van Damme, revealed that most were drilled in the anterior half of the shell (79.2, 61.7, and 80.6%, respectively; *n* = 96, 149, and 170 shells, respectively). Assessing shell thickness at a standardized location on the shell also controlled for differences in mussel length. Photographs of the cross sections were taken by placing the cross-section of the mussels flat on a scanner (model: CanoScan LiDE 110). Thickness was estimated using imaging software (ImageJ; Java 1.8.0_172) as the average of the maximum and minimum thicknesses of the cross-section disregarding the dorsal hinge and ventral growing lip as these regions are known to be thicker and thinner, respectively (Fig. S1).

Given that the thickness of a mussel varies along the anterior–posterior axis, the largest mussel (in shell length) drilled by each dogwhelk did not always contain the deepest drill hole. Thus, the largest several mussels for each dogwhelk were cut through the drill holes or partial drill holes on a bandsaw. Photographs of these complete or partial drill holes were then taken by placing the cross-section flat on a scanner. The depth of each drill hole was then assessed using image analysis software (ImageJ; Java 1.8.0_172). Dogwhelks that did not drill any mussels during the entire experiment were assigned a maximum depth of 0 mm.

### Spatial and temporal comparison of mussel shell thickness

Archived and contemporary *Mytilus californianus* mussel shells were used to determine if there was a consistent or variable mosaic of mussel shell thickness across space and time. Mussels were from three time periods (2000–2001, 2008–2009, and 2019), across the six study sites (Fig. 1). At each site, mussels were collected from very wave-exposed, mid-intertidal mussel beds where *N. canaliculata* were abundant. Across the three survey periods, mussels were collected from the same mussel bed at each site. Mussels were initially collected for several different projects, so the number of mussels and range of shell lengths for each site and time period differed slightly (see additional details in Section S2 and Table S1). For the contemporary samples used in this study, ~ 100 live mussels were collected from each site in 2019. The tissue was removed, and the shells were rinsed in fresh water and then air dried.

All mussels were cut at 1/3 the length of the left valve from the anterior end on a bandsaw, the cross-section was scanned, and the photos were used to calculate thickness via imaging software (ImageJ; Java 1.8.0_172) of the anterior section of the cut (see additional details in Section S2). We calculated thickness by averaging the maximum and minimum thicknesses measured along the cross-section, disregarding the dorsal hinge for maximum thickness and growing lip for minimum thickness, as these are known to be significantly thicker and thinner, respectively (Figure S1). We used the 2019 mussels to test whether our measurements of mean thickness showed comparable patterns to data from alternative shell thickness approaches. For all 2019 shells, indirect shell thickness across the entire left valve was calculated as: 1000* valve dry weight/surface area (where surface area = length × (height^2^ + width^2^)^0.5^ × π/2) (Freeman and Byers 2006). To evaluate a third method, we also used 2019 mussels from Soberanes Point and Strawberry Hill to determine the integrated thickness of the cross-section, measured as the area of the polygon of the scanned image divided by the length of the curved mussel shell (Fig. S1).

### Association between dogwhelk drilling capacity and mussel shell thickness

To test if there is a relationship between predator foraging ability and prey morphological traits, we compared dogwhelk drilling ability across the six sites to mussel shell thickness measured in the prior decade. To do this, we used two sets of data on dogwhelk drilling ability. The first was a previously published dataset from 2009 on dogwhelk drilling ability from the same six populations (Sanford and Worth 2009). This dataset measured drilling ability as a binary trait on a 50–70 mm mussel (i.e., whether a dogwhelk was capable of drilling at least one mussel of this size during a 100-day trial). Thus, for this analysis, we converted our 2019 data from the laboratory scoring experiment to a similar binary dataset (i.e., if the maximum size of mussel drilled per dogwhelk was beyond the 60 mm (± 10 mm) size class, the dogwhelk was classified as being able to drill a 50–70 mm mussel). Drilling success was quantified for each population and time period at the level of the family.

Current traits are a reflection of past selection; thus, we compared a dogwhelk’s drilling ability to mussel shell thickness measured in the prior decade. We compared the 2009 dogwhelk drilling ability to mussel shell thickness data from 2000 to 2001, and the 2019 dogwhelk drilling ability to the mussel shell thickness data from 2008 to 2009. The duration of the time-lag between changes in shell thickness and changes in drilling ability is unknown and is likely influenced by multiple factors, including genetic variation within dogwhelk populations. However, as an initial investigation of this question, we used the data at hand that covered multiple decades and populations distributed over ~ 1000 km.

### Analyses

All statistical analyses were carried out in R (version 3.5.1; R Core Team 2022). Linear regression was used to assess the correlation between mussel shell thickness and mussel length for the size classes used in the laboratory experiment; to fit assumptions, shell thickness was square root transformed. Welch’s two-sample t test was used to compare the shell thickness of the 150 mm mussel size class from the two populations, Bodega Marine Reserve and Strawberry Hill. We ran linear mixed-effects models to analyze if maximum mussel size drilled and maximum drill hole depth varied among the six dogwhelk populations. For these models, we treated site as a fixed effect and family as a random effect, since families are nested within site. Normal distribution of residuals was assessed with Q–Q plots and homoscedasticity was assessed using scale–location plots. When main effects were significant, we subsequently performed Tukey’s honestly significant difference (HSD) post hoc tests. Growth rate (log of change in dogwhelk length + 1) was analyzed using a linear mixed model with family as a random effect and site, sex, and their interaction as fixed effects. All mixed-effects models were run using the R packages “lmer” and “emmeans”. Analyses of whether the deepest drill hole was on a drilled versus partially drilled mussel were completed using Pearson’s chi-squared test.

To test for differences in mussel thickness across space and time, we used a linear model with site, time period, and their interaction as fixed effects. To control for the effect of mussel length on mussel shell thickness, we used the ratio of mean thickness at 1/3 the length of the mussel to mussel length as the response variable. The log of the ratio was used to fulfill assumptions of normality and homoscedasticity. Main effects were analyzed with an ANOVA. We performed post hoc tests on all levels of site within time period and time period within site with a Bonferroni correction to account for multiple comparisons. Integrated thickness measurements, across the entire left valve and across the entire cross-section, were compared to mean thickness using model II major axis (MA) linear regression using the R package “lmodel2” (Legendre 2018).

We tested the hypothesis that a dogwhelk’s drilling ability was related to mussel shell thickness measured in the prior decade. We performed model II reduced major axis (RMA) linear regression analyses using the R package “lmodel2” on mean family-level dogwhelk drilling success and model estimates of shell thickness across the six sites.

## Results

### Variation in drilling capacity among populations

Mussel length was a reliable indicator of shell thickness, confirming that presenting dogwhelks with a sequence of increasing mussel sizes challenged individuals to drill thicker mussels. The square root of average thickness increased linearly with mussel length across the eight mussel size classes (Fig. S2; linear regression, *t*_158_ = 34.51, *P* < 0.001, slope = 0.008, *r*^2^ = 0.882). The thickness of 150 mm mussels from the two populations were not different from each other (Welch’s two sample *t* test, *t*_33_ = 1.861, *P* = 0.072).

**Fig. 2:**
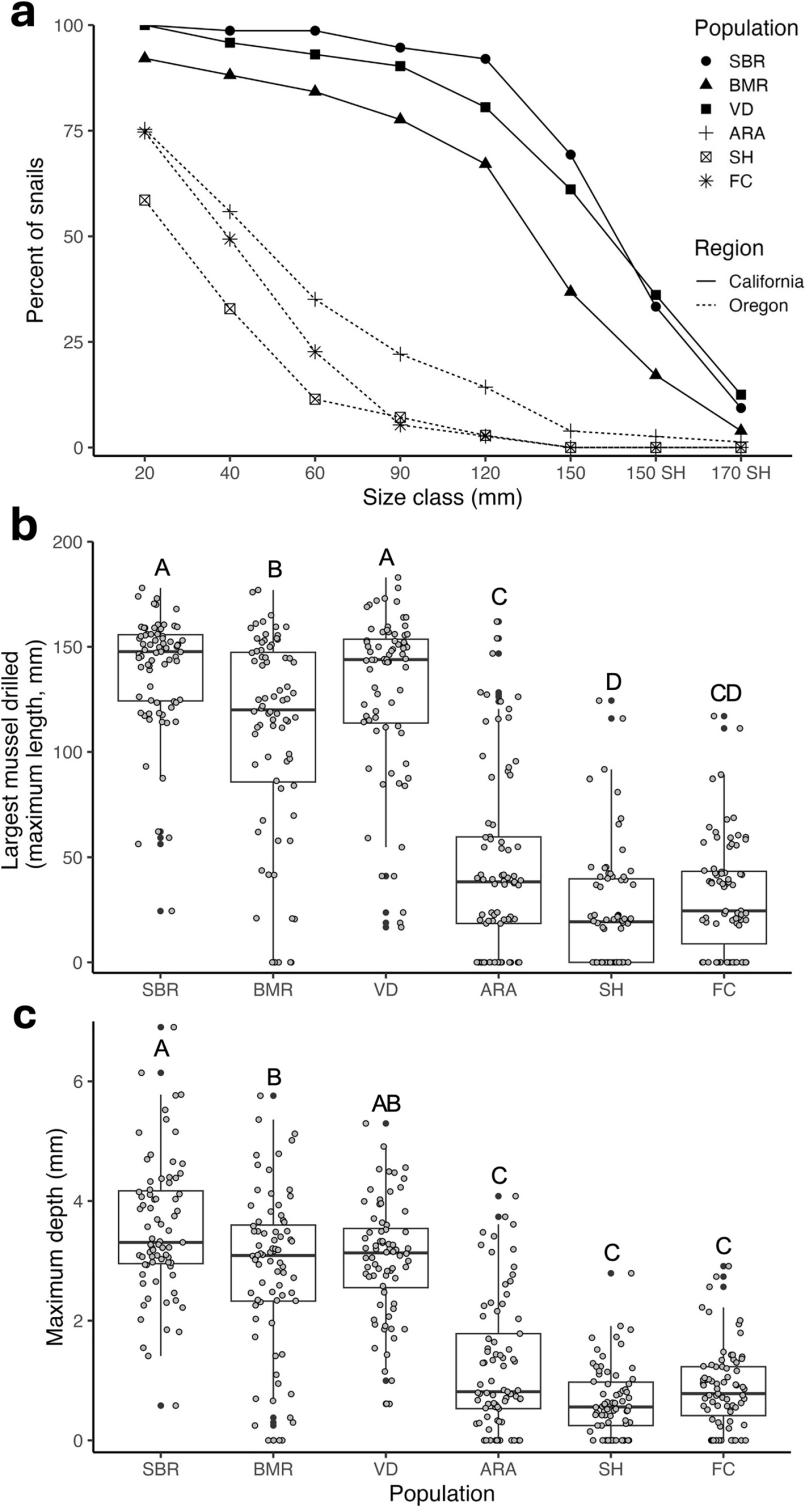
Variation in drilling capacity among six *N. canaliculata* populations assessed in the laboratory (*n* = 445). **a** Percentage from each population that drilled each mussel size class. All mussels were collected from Bodega Marine Reserve, except “150SH” and “170SH” were collected from Strawberry Hill. Drilling capacity of dogwhelks was quantified as **b** the largest mussel drilled (maximum length), and **c** maximum drill hole depth. Shared letters indicate populations that are not significantly different (Tukey HSD, *P* > 0.05). See Fig. 1 for site abbreviations

California and Oregon dogwhelk populations were strikingly divergent in the maximum size of mussels that dogwhelks could drill successfully. The separation between regions was apparent at the smallest size class, where almost all California dogwhelks drilled a 20 mm mussel, while only 57–73% of Oregon dogwhelks drilled a mussel of this size (Fig. 2a). The separation between dogwhelks from the two regions was even greater for larger mussels. The maximum sized mussel drilled differed significantly among populations (Fig. 2b; one-way ANOVA, *F*_5,41.75_ = 115.5, *P* < 0.001), with dogwhelks from California populations drilling larger mussels than dogwhelks from Oregon. The Soberanes Point and Van Damme populations stood out as having the greatest capacity to drill large mussels. Based on model estimates, dogwhelks from these two populations drilled mussels that were on average 4.8 times larger than the largest mussels drilled by dogwhelks from Strawberry Hill and Fogarty Creek, the two weakest drilling populations.

As expected, results for maximum drill hole depth paralleled those for largest mussel drilled. Maximum drill hole depth differed significantly among populations (Fig. 2c; one-way ANOVA, *F*_5,41.85_ = 71.238, *P* < 0.001). Based on model estimates, dogwhelks from California had the capacity to produce drill holes that were on average 3.4 times deeper than the drill holes produced from Oregon dogwhelks.

For some dogwhelks, their maximum drill hole depth was from mussels that were only partially drilled (Table S2). This scenario was much more common for the populations from Oregon than California (Pearson’s Chi-squared test, *χ*^2^ = 37.674, df = 5, *P* < 0.001). Across all populations, the mussel that contained the maximum drill hole depth was most often (52–70% of individuals) from the largest mussel drilled (Table S3). For those situations that differed, dogwhelks from California frequently (31–39% of individuals) were able to drill a mussel that was longer than the mussel that contained the deepest drill hole, usually by drilling through more posterior, thinner regions of very large mussel shells. In comparison, dogwhelks from Oregon often (26–32% of individuals) had partial drill holes on mussels that were larger than the maximum sized mussel drilled, suggesting that they tried unsuccessfully to drill thick shells that exceeded their drilling capacity.

Over the course of the 25-week experiment, there was substantial growth in the dogwhelks. Growth (log of change in length + 1) differed among the six populations (Fig. S3; two-way ANOVA, *F*_5,42.04_ = 187.024, *P* < 0.001) with the greatest increase in the three California populations. Although drilled mussels were removed before dogwhelks could completely consume their prey, differences in drilling ability still resulted in higher consumption and growth in California dogwhelks. Female dogwhelks grew more than male dogwhelks (Fig. S3; two-way ANOVA, *F*_1, 432.78_ = 144.324, *P* < 0.001), and the growth rate of the two sexes differed among the six sites (two-way ANOVA, “sex*site”, *F*_5,431.74_ = 9.118, *P* < 0.001).

### Spatial and temporal variation in mussel shell thickness

Mean mussel shell thickness at 1/3 the mussel length was tightly correlated with the integrated shell thickness across the entire left valve (Fig. S4) for all 2019 mussel shells and the integrated thickness across the entire cross-section for the 2019 Soberanes Point and Strawberry Hill populations (Fig. S5). Thus, we report mean shell thickness as our metric for the remaining analyses.

**Fig. 3:**
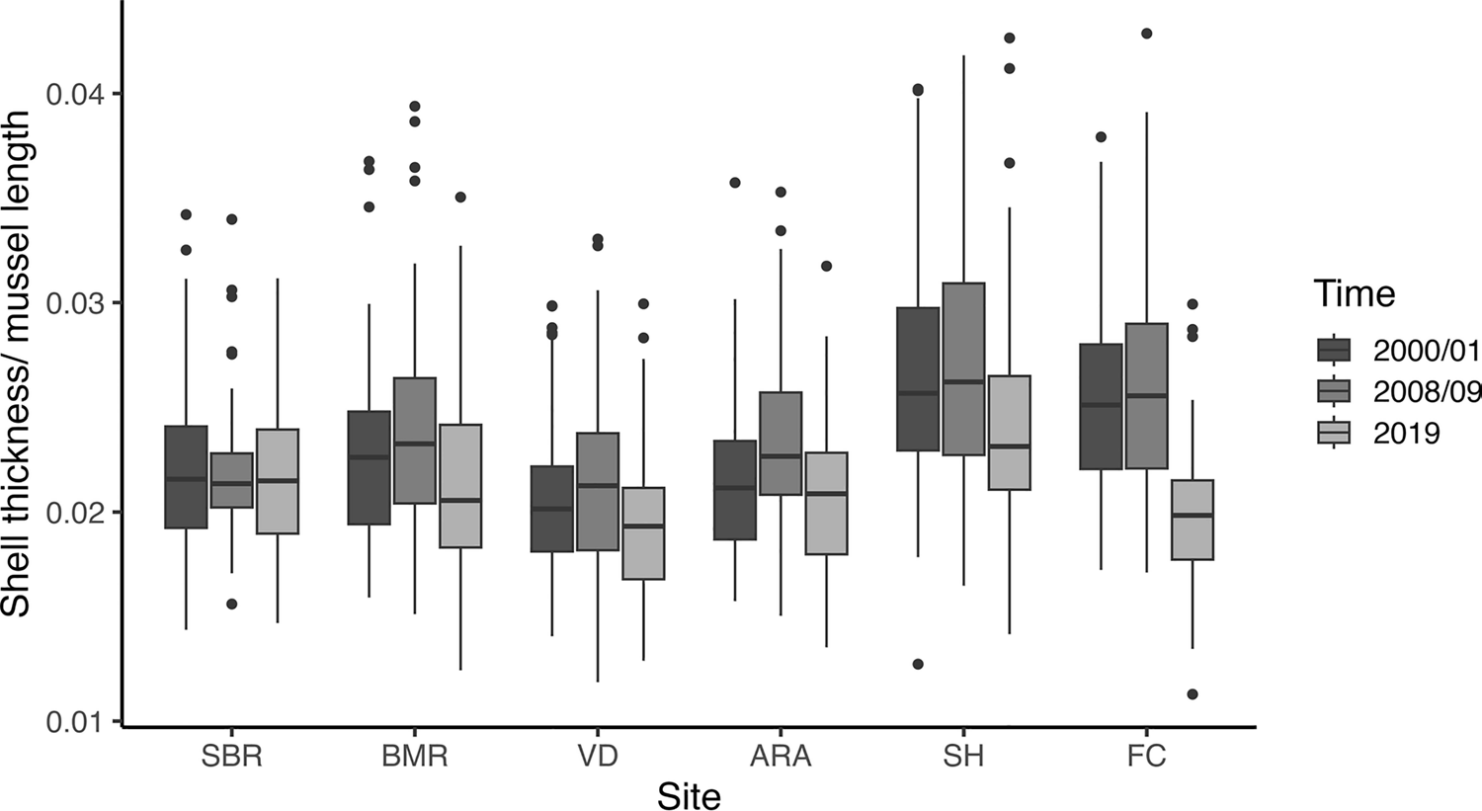
Variation in the ratio of shell thickness to length for the mussel *Mytilus californianus* (*n* = 1913). Box plots show differences among the six sites and three sampling time periods. Sites are ordered from South to North along the x-axis. The log of the ratio of mussel shell thickness to length varied significantly with site, time period, and their interaction (ANOVA; site *F*_5,1895_ = 67.8, *P* < 0.001; time period *F*_2,1895_ = 71.8 *P* < 0.001; site*time period, *F*_10,1895_ = 7.21, *P* < 0.001). See text for additional statistical results. See Fig. 1 for site abbreviations

To determine spatial and temporal patterns of mussel shell thickness over a 20-year period, we analyzed the log of the ratio of mean mussel shell thickness at 1/3 the length of the mussel to mussel length. Using this ratio allowed us to control for differences in the lengths of mussels sampled among sites and time periods, since our laboratory experiment showed that mussel thickness increases with mussel length (Fig. S1). In the full model, mussel shell thickness varied significantly with site, time period, and their interaction (ANOVA; site, *F*_5,1895_ = 67.790, *P* < 0.001; time period, *F*_2,1895_ = 71.767 *P* < 0.001; site*time period, *F*_10,1895_ = 7.211, *P* < 0.001). For the 2000–2001 and 2008–2009 time periods, the two central Oregon sites (SH and FC) had mussels that were 1.20 times and 1.18 times thicker than those from the other sites, respectively (Fig. 3; Table S4). The northern California site of Van Damme had the thinnest mussels in both 2000–2001 and 2008–2009 (Table S4). In 2019, the spatial pattern changed; the thickest mussels were still found at Strawberry Hill, while the thinnest mussels occurred at Fogarty Creek and Van Damme (Table S4). When analyzing each site separately, there are clear temporal patterns of mussels thinning over time (Fig. 3). Mussel shells at all sites, except Soberanes Point, were thinner in 2019 than 2008–2009 (Table S5). Furthermore, both central Oregon sites were 1.18 times and 1.21 times thinner in 2019 than 2000–2001 and 2008–2009, respectively (Table S5).

### Association between dogwhelk drilling capacity and mussel shell thickness

**Fig. 4:**
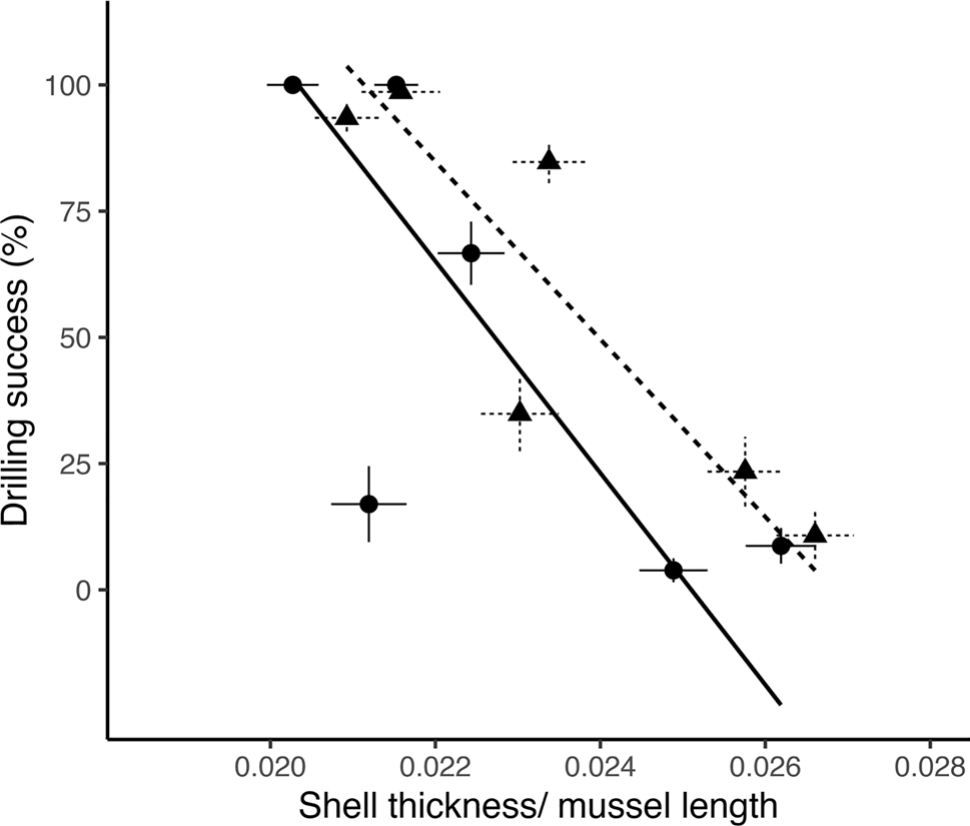
Relationship between drilling capacity of *N. canaliculata* and the thickness of mussels (*M. californianus*) from the previous decade. Data plotted are model estimates and standard error for each of the six sites for the mussel thickness model from 2000 to 2001 and 2008 to 2009, and mean family-level percent drilling success (i.e., capacity to drill at least one 50–70 mm-long mussel during the laboratory trial) and standard error for each of the six dogwhelk populations (2009 data are from Sanford and Worth 2009 and 2019 data presented here). Lines are model II reduced major axis (RMA) regression lines showing significant and suggestive relationships. Circles and the solid line represent the 2000–2001 shell thickness data and 2009 dogwhelk drilling traits (*P* = 0.10, *r*^2^ = 0.53). Triangles and the dotted line represent the 2008–2009 shell thickness data and 2019 dogwhelk drilling traits (*P* = 0.020, *r*^2^ = 0.78)

The drilling capacity of dogwhelks from each population was related to past mussel shell thickness at that site (Fig. 4; Table S6). There was suggestive evidence of a negative relationship between 2000 and 2001 mussel shell thickness and 2009 drilling success (model II RMA regression, *P* = 0.100, slope = − 26,397.91, *r*^2^ = 0.532). For the 2008–2009 mussel shell data and 2019 drilling success, there was a significant negative relationship (model II RMA regression, *P* = 0.020, slope = −19,451.86, *r*^2^ = 0.778). These negative relationships suggest that populations of dogwhelks with stronger drilling capacity were associated with thinner mussels.

## Discussion

Our findings document a striking mosaic of geographic variation in the interaction between a drilling marine dogwhelk, *N. canaliculata*, and its mussel prey, *Mytilus californianus*. Dogwhelks from California populations can drill mussels that are substantially larger and thicker than dogwhelks from Oregon. This spatial mosaic of drilling capacity is associated with variation in mussel shell thickness, a prey defense known to strongly influence foraging strategies in predatory dogwhelks (Kroeker et al. 2016; Sherker et al. 2017). However, the potential role of selection imposed by spatial variation in shell thickness may have changed over time with recent mussels being thinner than a decade prior, particularly on the central Oregon coast.

### A spatial mosaic of selection

Our results are consistent with the hypothesis that spatial variation in shell thickness has contributed to adaptive differentiation among populations of dogwhelks. *M. californianus* shells have been consistently thinner in California than in Oregon over time (Fig. 3). A greater drilling capacity in dogwhelk populations in California (Fig. 2) is associated with thinner shells in this region (Fig. 4), a pattern that may seem counterintuitive. However, we hypothesize that these thinner shells may have initially led *N. canaliculata* in California to target *M. californianus* as a viable prey item. Over time, selection to prey on larger mussels with greater tissue mass and thicker shells favored the continued evolution of greater drilling capacity in dogwhelks from California. In contrast, we hypothesize that thicker *M. californianus* shells in Oregon dissuaded dogwhelks in this region from preying on this species. In addition, previous research has shown that the abundance of alternative prey (including barnacles and thin-shelled *M. trossulus* mussels) is greater in Oregon than California (Broitman et al. 2008), which might have further weakened selection pressure on Oregon dogwhelk populations (Sanford and Worth 2010). Thus, our findings are consistent with the hypothesis that the strength of selection varies with prey defenses across the overlapping geographic ranges of these two species. In particular, there may be strong prey-driven selection on the predator in California, where thinner shells and increased prey profitability selected for greater drilling capacity as dogwhelks exploited larger mussels. In contrast, thicker mussels may lead to a selection cold spot on the central Oregon coast.

Previous research has linked spatial variation in *M. californianus* growth and shell properties to oceanographic conditions, including seawater temperature, phytoplankton concentrations, and pH (Menge et al. 2008; Kroeker et al. 2016). However, research in the Pacific Northwest and northern California suggests that ocean acidification associated with anthropogenic climate change is altering mussel shell calcification (Pfister et al. 2016; Vriesman et al. 2022). Comparisons of the mineralogical composition of archival shell collections versus modern counterparts suggest that *M. californianus* are calcifying less and are thinner than mussels in the past decade and millennia, linking these declines to oceanographic variability and declining seawater pH (Pfister et al. 2016; Vriesman et al. 2022). Our analyses build on this growing body of work and document a pattern of mussel thinning at sites in Oregon and California over the past two decades. Most notably, sites on the central Oregon coast, which previously had the thickest mussels, experienced the greatest declines in shell thickness. These changes may result in a shift in the adaptive landscape that influences drilling phenotypes in *N. canaliculata* populations. If this trend continues, *M. californianus* on the central Oregon coast may become thin enough to serve as viable prey for dogwhelks. Such a shift could drive substantial ecological changes in these rocky shore communities, as mid- to large-sized *M. californianus* have historically been largely immune to dogwhelk predation at most sites in Oregon (Sanford and Worth 2009, 2010).

Despite these broad regional generalities, there are population-level differences in drilling capacity within regions, and thus, this adaptive landscape is best characterized as a mosaic rather than a uniform gradient. For example, dogwhelks from Soberanes Point and Van Damme stand out as the two populations with the greatest drilling capacity. These populations are separated by 430 km of coastline with populations of moderate drillers interspersed (e.g., BMR; Fig. 2). *N. canaliculata* has direct development and very limited dispersal, and previous research has shown that genetic variation among populations along the coast displays a pattern of isolation by distance (Sanford et al. 2003). Thus, these populations likely evolved in isolation, creating a promising system to explore future questions about parallel evolution of adaptive phenotypes.

### Mechanisms underlying geographic variation in predator phenotypes

The mechanisms underlying the observed variation in drilling capacity (i.e., maximum drill hole depth) among populations of *N. canaliculata* remain to be determined. Drilling by predatory gastropods is a chemo-mechanical process in which the accessory boring organ (ABO) and radula alternate between secreting acid and scraping away the weakened shell, respectively (Carriker 1981). Drilling through a thick mussel is a slow process (estimated rate = 0.29 mm/day for the congener *Nucella lapillus*; Rovero et al. 1999) and thus represents a substantial investment of time and energy. The secretory disk of the ABO is mounted at the end of an extensible stalk. As the dogwhelk drills a progressively deeper hole, it must extend the ABO stalk to the bottom of the hole for the disk to secrete acid. Thus, the length of the ABO stalk may limit the thickness of mussel shell that can be drilled (Carriker and Van Zandt 1972). Future anatomical and gene ontology studies are needed to explore whether variation in the length of the ABO stalk or other biochemical traits might underlie population-level differences in drilling capacity.

We cannot rule out that behavioral components might also contribute to the observed variation among dogwhelk populations in drilling capacity (Foster 1999; Neylan et al. 2024). To identify the maximum sized mussel drilled, we gave dogwhelks mussels of increasing size from primarily one source location. Thus, those dogwhelks that progressed further along the series of mussel size classes had more handling experience, which may have prepared them for the progressively larger prey items. Previous studies have yielded contrasting results when testing whether individuals with experience handling a specific prey item learn to identify preferred drill site locations and optimal prey size (Hughes and Dunkin 1984; Palmer 1984; Hart and Palmer 1987). As dogwhelks from California progressed through the mussel size classes, they also grew larger (Fig. S3), which may have increased their ability to target larger mussels (e.g., Hughes and Dunkin 1984; Chiba and Sato 2012). However, the population-level differences in drilling became apparent even at the small mussel size classes (20, 40, and 60 mm), when dogwhelks from all populations were of similar size. Whether or not behavior plays a role in these patterns, the differences that we documented among dogwhelk populations in the maximum size of mussels drilled closely parallel those observed in field surveys of drilling predation in mussel beds at these same sites (Sanford and Worth 2009). Moreover, previous work on *N. canaliculata* that raised dogwhelks through two generations on a common diet in the laboratory has shown that there is a genetic basis to variation in drilling capacity (Sanford and Worth 2009), suggesting that selection has shaped the morphological and/or behavioral mechanisms underlying geographic variation in drilling predation.

### Ecological consequences of divergent predator phenotypes

The divergent evolution of drilling across part of the species range likely has strong eco-evolutionary effects, such that this intertidal predator may serve different functional roles across its biogeographic range. We hypothesize that *N. canaliculata* in California may have greater community effects than in Oregon, due to their stronger impacts on the foundation species *M. californianus* (Fig. 2). Previous work showed that *N. canaliculata* in California drilled ~ 19% of mussels transplanted into the low intertidal zone over 9 months, compared to zero mussels in Oregon despite higher natural dogwhelk densities (Sanford et al. 2003). In contrast to the sea star *Pisaster ochraceus*, which has been shown to be a size limited predator (Paine 1976), we have shown that *N. canaliculata* from populations in California can drill mussels 17 cm in length, which represent the largest and oldest mussels found in mussel beds. Individual dogwhelks typically do not move far and often drill adjacent mussels creating patches of large, empty shells that remain within the bed (Sanford and Worth 2009). This higher occurrence of drilling, particularly of large mussels, may weaken the strength of the mussel matrix increasing the likelihood of dislodgement during a disturbance (Brosnan 1994). Space is often the primary limiting resource on temperate rocky shores, and disturbance to mussel beds drives succession and the spatial patterning of organisms in this community (Sousa 1985). In contrast, *N. canaliculata* may have a smaller influence on rocky shore community dynamics in Oregon. This is in line with previous research on the central Oregon coast showing that dogwhelks have weaker effects than *P. ochraceus* (Cerny-Chipman et al. 2017), preying mostly on barnacles and *M. trossulus* or small *M. californianus* (Sanford and Worth 2010). Although understudied, spatial variation in predator phenotypes is likely important for understanding food web structure and eco-evolutionary dynamics in both marine and terrestrial ecosystems (Reznick and Travis 2019; Urban et al. 2020).

### Potential role of dispersal and temporal variation in shaping mosaics of selection

Intense drilling predation pressure exerted by *N. canaliculata* in California might be expected to impose reciprocal selection for increased shell thickness in *M. californianus*. We observed no evidence to support this hypothesis; indeed, *M. californianus* shells are thinnest at California sites where drilling predation is the most intense (Fig. 4). Exposure to waterborne cues from predatory dogwhelks has been found to induce a plastic response of shell thickening in some mussel species (Sherker et al. 2017). However, we are not aware of any evidence that exposure to *N. canaliculata* cues induces plastic shell thickening in *M. californianus*.

The apparent lack of reciprocal selection on the prey species in this interaction might be influenced by differences in life history and dispersal potential (Wieters et al. 2008). Mismatches in dispersal among interacting species can result in unique selection mosaics for coevolving species with consequences for understanding local adaptation and eco-evolutionary dynamics (Vogwill et al. 2008; Urban et al. 2020). For example, a weevil that preys on seeds has a much greater dispersal potential than its host plant, the Japanese camellia, leading to a homogenizing effect that promotes evolutionary convergence among populations of the coevolving plant (Toju et al. 2011). In contrast, in our study system, the prey species (*M. californianus*) has high dispersal potential and high gene flow across its geographic range (Addison et al. 2008), whereas the predator (*N. canaliculata*) lacks planktonic dispersal and is highly differentiated among sites (Sanford et al. 2003). These differences in dispersal potential and the asymmetrical strength of selection pressures on predator and prey may lead to a system where spatial variation in prey traits (e.g., shell thickness) is environmentally driven, while low dispersal in the predator promotes local adaptation in consumer phenotypes, with broader ecological consequences for the surrounding community.

Adaptive landscapes are usually not static in time. Quantifying temporal changes in selection is important for understanding ecological and evolutionary patterns in communities. Anthropogenically driven climate change is modifying both biotic and abiotic environments and will have consequences for almost all species interactions (Tylianakis et al. 2008; Kroeker and Sanford 2022). In marine ecosystems, ocean acidification is affecting mussel shell calcification along the Pacific Coast of North America (Pfister et al. 2016; Vriesman et al. 2022), and our findings are consistent with this pattern (Fig. 3). This changing landscape of mussel shell thickness may impact prey-driven selection on populations of the dogwhelk *N. canaliculata*. However, the capacity and rate at which Oregon populations of *N. canaliculata* might evolve to target *M. californianus* will depend on the level of standing genetic variation present in populations. A greater understanding of gene flow in this species and population structure along the coast is needed to predict how this species may or may not adapt as conditions change. Possibly, this shifting selection landscape may change the role that this predator plays in rocky shore community dynamics. Overall, our results highlight the importance of studying predator–prey interactions within a geographic context of varying environmental pressures. These forces likely play an underappreciated role in dictating evolutionary patterns of phenotypic divergence, with ecological consequences for communities in a rapidly changing world.

## Supplementary Information

Below is the link to the electronic supplementary material.

Supplementary file1 (DOCX 1098 KB)

## Data Availability

The datasets are archived at Biological and Chemical Oceanography Data Management Office (BCO-DMO): https://www.bco-dmo.org/project/811409.

## Code availability

Code and datasets to replicate analyses are published to Zenodo (https://doi.org/10.5281/zenodo.11038182).
