# Supplementary material for: Biogeographic variation in mussel shell thickness and drilling predation on rocky shores
Source: Oecologia. 2025 Jul 8;207(8):126. doi: 10.1007/s00442-025-05760-x (PMC12238184; doi:10.1007/s00442-025-05760-x)
Supplement: Supplementary file 1 — Supplementary file1 (DOCX 1098 KB) [file 442_2025_5760_MOESM1_ESM.docx]

**Supplemental Material:**

Biogeographic variation in mussel shell thickness and

drilling predation on rocky shores

Emily K. Longman^1,2,†^ & Eric Sanford^1,2^

^1^ Bodega Marine Laboratory, University of California Davis, Bodega Bay, CA, 94923, USA

^2^ Department of Evolution and Ecology, University of California Davis, Davis, CA, 95616, USA

†Present Address: Department of Biology, University of Vermont, Burlington, VT, 05405, USA

Corresponding Author: Emily K. Longman, Emily.Longman@uvm.edu

**Section S1** Additional Details on Laboratory Scoring Experiment

Dogwhelks were given mussels of increasing size and thickness to quantify their maximum drilling capacity. For mussel size classes less than 150mm, each snail was given two mussels, one slightly less and one slightly more than the mean for that size class. For mussel size classes greater than 150mm, snails were only given one mussel. The time between experimental checks during the 25-week experiment was set to be long enough to reduce disturbance to the snails during the drilling process, but short enough to prevent snails from consuming all of the tissue in the mussels they had drilled. The first two checks were two weeks apart, the next three checks were three weeks apart, and the final three checks were four weeks apart. Snails that died during the first 7 weeks (N = 9), were replaced with another snail from the same family and started at the beginning of the series of mussel size classes.

At the start of the experiment, all snails were similar in size (range of snail lengths: 18-22mm). During the period when snail feeding rates were standardized on a diet of 10 *M. trossulus*, the dogwhelks all grew slightly (mean = 21.5mm, range = 18.6 - 23.5mm, One-way ANOVA, population, F_5,42_ = 2.175, *P* = 0.075).

**Section S2** Additional Methodological Details on Spatial and Temporal Comparisons of Mussel Shell Thickness

The archived (2000-2001 and 2008-2009) mussel shells used in the temporal and spatial comparison of shell thickness were collected for several different projects, so the number and range of shell lengths varied slightly for each site and time period (Table S1). The 2000-2001 mussel shells came from three surveys. First, in 2000, approximately 100 empty mussel shells (some drilled, some undrilled) were collected from within the mussel beds at Soberanes Point. Live mussels representing a similar size range from Strawberry Hill and Fogarty Creek were subsequently collected in 2000. The tissue was removed from the live mussels and all shells were air dried. Second, in 2001, 25 live mussels from a restricted size range (55-73mm long) were collected from each site (except for Cape Arago, where 50 mussels were collected). Tissue was removed from the mussels and the shells were air dried. Lastly, in 2001, quadrat surveys of drilled mussels were completed at a subset of the sites (SBR, BMR, VD, ARA, plus 3 mussels from FC). The 2008-2009 mussel shells were originally collected to survey predation in mussel beds (Sanford & Worth 2009). At each site, mussels from ten 0.25m^2^ quadrats were removed, measured, and assessed for drill holes.

The mussels analyzed were collected over several decades for projects with different goals, so there was some variation in the collection and processing of samples. First, some of the mussels in the 2000-2001 and 2008-2009 time periods were drilled, and others were collected live. However, *Nucella canaliculata* cannot differentiate between mussels of the same size that differ in shell thickness (Quan & Longman, unpublished data), so we assume that either drilled or undrilled mussels were representative of a typical shell thickness present during that time interval. Second, although we tried to analyze the left valve for consistency, some of the 2008-2009 mussels were previously cut on the right valve and the intact left valve was unavailable for our analyses. Third, after cutting shells at 1/3 their length, we generally analyzed the resulting cross section on the anterior piece of the shell. However, some of the 2000-2001 and 2008-2009 shells had to be analyzed on the posterior cross section due to missing pieces as these sections had been cut for previous experiments. The effects of variation in these aspects of shell processing were analyzed with linear models with shell length as a covariate and either valve (left vs. right) or shell fragment scanned (anterior vs. posterior) as a fixed factor. Shell thicknesses of the 2008-09 archived shells did not vary significantly between left versus right valves (ANCOVA; shell length, F_1, 538_ = 388.580, *P* < 0.001; valve, F_1, 538_ = 0.137, *P* = 0.711) nor between the anterior versus posterior mussel section (ANCOVA; shell length, F_1,538_ = 388.768, *P* < 0.001; fragment scanned, F_1,538_ = 0.399, *P* = 0.528). Lastly, if archived shells were worn (i.e., the periostracum was completely missing) they were excluded from our analysis.

**Table S1** Sample sizes and range of lengths of mussels (*Mytilus californianus*) in mm collected across the three time periods and six sites. See Fig. 1 for site abbreviations.

| **Time Period** | **Site** | **Number of Mussels** | **Mussel Length Range (mm)** |
| --- | --- | --- | --- |
| 2000/01 | SBR | 214 | 17.02 – 123.80 |
|  | BMR | 100 | 24.07 – 115.07 |
|  | VD | 137 | 21.38 – 126.13 |
|  | ARA | 70 | 19.03 – 104.30 |
|  | SH | 122 | 17.54 – 131.49 |
|  | FC | 118 | 32.27 – 126.81 |
| 2008/09 | SBR | 67 | 53.03 – 110.40 |
|  | BMR | 92 | 50.38 – 123.86 |
|  | VD | 95 | 50.43 – 140.69 |
|  | ARA | 78 | 46.54 – 119.12 |
|  | SH | 105 | 49.21 – 124.30 |
|  | FC | 104 | 51.16 – 124.48 |
| 2019 | SBR | 104 | 49.28 – 125.42 |
|  | BMR | 101 | 49.61 – 126.93 |
|  | VD | 104 | 51.94 – 157.80 |
|  | ARA | 93 | 49.03 – 138.01 |
|  | SH | 108 | 43.41 – 136.32 |
|  | FC | 101 | 50.37 – 131.67 |

**Table S2** Percent of occasions where maximum drill hole depth per snail was recorded on a mussel that was partially drilled versus completely drilled (n = 70-77 snails per population). See Fig. 1 for site abbreviations.

| **Snail Population** | **Partial Drill Hole** (%) | **Complete Drill Hole** (%) |
| --- | --- | --- |
| SBR | 8.00 | 92.00 |
| BMR | 26.32 | 68.42 |
| VD | 11.11 | 88.89 |
| ARA | 31.17 | 53.25 |
| SH | 30.00 | 47.14 |
| FC | 34.67 | 48.00 |

**Table S3** Assessment among 6 populations of *Nucella canaliculata* of whether the maximum drill hole depth recorded for each snail was on the longest mussel drilled successfully. Possibilities were: (a) the deepest drill hole recorded was a partial drill hole on a mussel longer than the longest mussel drilled successfully; (b) the deepest drill hole recorded was on the longest mussel drilled successfully; (c) the deepest drill hole recorded was on a mussel smaller than the longest mussel drilled successfully. See Fig. 1 for site abbreviations.

| **Snail Population** | **Deepest drill hole was a partial drill hole on a mussel longer in length than longest mussel drilled** (%) | **Deepest drill hole was on longest mussel drilled** (%) | **Deepest drill hole was on a mussel smaller in length than longest mussel drilled** (%) |
| --- | --- | --- | --- |
| SBR | 4.00 | 62.67 | 33.33 |
| BMR | 15.79 | 52.63 | 31.58 |
| VD | 5.56 | 55.56 | 38.89 |
| ARA | 25.97 | 70.13 | 3.90 |
| SH | 30.00 | 62.86 | 7.14 |
| FC | 32.00 | 60.00 | 8.00 |

**Table S4** Mussel shell thickness model estimates across the 3 time periods and 6 sites. Model was performed on the log scale, but estimates are back transformed. See Fig. 1 for site abbreviations.

| **Time Period** | **Site** | **Model Estimate** | **Standard Error** |
| --- | --- | --- | --- |
| 2000-2001 | SBR | 0.021526 | 0.000265 |
|  | BMR | 0.022433 | 0.000405 |
|  | VD | 0.020271 | 0.000312 |
|  | ARA | 0.021193 | 0.000457 |
|  | SH | 0.026190 | 0.000428 |
|  | FC | 0.024887 | 0.000413 |
| 2008-2009 | SBR | 0.021580 | 0.000475 |
|  | BMR | 0.023377 | 0.000440 |
|  | VD | 0.020926 | 0.000387 |
|  | ARA | 0.023024 | 0.000470 |
|  | SH | 0.026604 | 0.000468 |
|  | FC | 0.025756 | 0.000455 |
| 2019 | SBR | 0.021371 | 0.000378 |
|  | BMR | 0.020842 | 0.000374 |
|  | VD | 0.019150 | 0.000339 |
|  | ARA | 0.020472 | 0.000383 |
|  | SH | 0.023537 | 0.000408 |
|  | FC | 0.019713 | 0.000354 |

**Table S5** Mussel shell thickness model contrasts across the 3 time periods and 6 sites. The model was performed on the log scale, but estimates are back transformed. Contrasts were performed for all sites within each time period and all time periods within each site. *P*-values are Bonferroni corrected. See Fig. 1 for site abbreviations.

| **Time Period** | **Site Code** | **Contrast** | **Ratio** | **Standard Error** | **t ratio** | ***P*-value** |
| --- | --- | --- | --- | --- | --- | --- |
| 2000/01 | . | SBR / BMR | 0.9596 | 0.0210 | -1.8900 | 1.0000 |
| 2000/01 | . | SBR / VD | 1.0619 | 0.0210 | 3.0445 | 0.1489 |
| 2000/01 | . | SBR / ARA | 1.0157 | 0.0252 | 0.6270 | 1.0000 |
| 2000/01 | . | SBR / SH | 0.8219 | 0.0168 | -9.5876 | 1.71E-19 |
| 2000/01 | . | SBR / FC | 0.8650 | 0.0179 | -7.0162 | 1.99E-10 |
| 2000/01 | . | BMR / VD | 1.1067 | 0.0262 | 4.2734 | 0.0013 |
| 2000/01 | . | BMR / ARA | 1.0585 | 0.0297 | 2.0230 | 1.0000 |
| 2000/01 | . | BMR / SH | 0.8565 | 0.0208 | -6.3658 | 1.53E-08 |
| 2000/01 | . | BMR / FC | 0.9014 | 0.0221 | -4.2344 | 0.0015 |
| 2000/01 | . | VD / ARA | 0.9565 | 0.0253 | -1.6798 | 1.0000 |
| 2000/01 | . | VD / SH | 0.7740 | 0.0174 | -11.4134 | 1.99E-27 |
| 2000/01 | . | VD / FC | 0.8145 | 0.0184 | -9.0578 | 2.03E-17 |
| 2000/01 | . | ARA / SH | 0.8092 | 0.0219 | -7.8296 | 5.09E-13 |
| 2000/01 | . | ARA / FC | 0.8516 | 0.0232 | -5.9047 | 2.63E-07 |
| 2000/01 | . | SH / FC | 1.0524 | 0.0245 | 2.1931 | 1.0000 |
| 2008/09 | . | SBR / BMR | 0.9231 | 0.0267 | -2.7617 | 0.3658 |
| 2008/09 | . | SBR / VD | 1.0313 | 0.0297 | 1.0703 | 1.0000 |
| 2008/09 | . | SBR / ARA | 0.9373 | 0.0282 | -2.1563 | 1.0000 |
| 2008/09 | . | SBR / SH | 0.8112 | 0.0229 | -7.4213 | 1.10E-11 |
| 2008/09 | . | SBR / FC | 0.8379 | 0.0237 | -6.2608 | 2.98E-08 |
| 2008/09 | . | BMR / VD | 1.1172 | 0.0295 | 4.1997 | 0.0018 |
| 2008/09 | . | BMR / ARA | 1.0153 | 0.0282 | 0.5482 | 1.0000 |
| 2008/09 | . | BMR / SH | 0.8787 | 0.0226 | -5.0199 | 3.56E-05 |
| 2008/09 | . | BMR / FC | 0.9077 | 0.0234 | -3.7536 | 0.0113 |
| 2008/09 | . | VD / ARA | 0.9089 | 0.0250 | -3.4682 | 0.0337 |
| 2008/09 | . | VD / SH | 0.7866 | 0.0201 | -9.4010 | 9.46E-19 |
| 2008/09 | . | VD / FC | 0.8125 | 0.0208 | -8.1140 | 5.49E-14 |
| 2008/09 | . | ARA / SH | 0.8655 | 0.0233 | -5.3601 | 5.88E-06 |
| 2008/09 | . | ARA / FC | 0.8940 | 0.0241 | -4.1500 | 0.0022 |
| 2008/09 | . | SH / FC | 1.0329 | 0.0258 | 1.2983 | 1.0000 |
| 2019 | . | SBR / BMR | 1.0254 | 0.0258 | 0.9949 | 1.0000 |
| 2019 | . | SBR / VD | 1.1160 | 0.0279 | 4.3886 | 0.0008 |
| 2019 | . | SBR / ARA | 1.0439 | 0.0269 | 1.6695 | 1.0000 |
| 2019 | . | SBR / SH | 0.9080 | 0.0225 | -3.8959 | 0.0064 |
| 2019 | . | SBR / FC | 1.0841 | 0.0273 | 3.2051 | 0.0865 |
| 2019 | . | BMR / VD | 1.0884 | 0.0274 | 3.3614 | 0.0498 |
| 2019 | . | BMR / ARA | 1.0181 | 0.0264 | 0.6908 | 1.0000 |
| 2019 | . | BMR / SH | 0.8855 | 0.0221 | -4.8709 | 7.58E-05 |
| 2019 | . | BMR / FC | 1.0573 | 0.0268 | 2.1943 | 1.0000 |
| 2019 | . | VD / ARA | 0.9354 | 0.0241 | -2.5948 | 0.6008 |
| 2019 | . | VD / SH | 0.8136 | 0.0202 | -8.3257 | 9.98E-15 |
| 2019 | . | VD / FC | 0.9714 | 0.0245 | -1.1512 | 1.0000 |
| 2019 | . | ARA / SH | 0.8698 | 0.0222 | -5.4678 | 3.25E-06 |
| 2019 | . | ARA / FC | 1.0385 | 0.0269 | 1.4578 | 1.0000 |
| 2019 | . | SH / FC | 1.1940 | 0.0298 | 7.1016 | 1.09E-10 |
| . | SBR | (2000/01) / (2008/09) | 0.9975 | 0.0252 | -0.1004 | 1.0000 |
| . | SBR | (2000/01) / 2019 | 1.0072 | 0.0217 | 0.3342 | 1.0000 |
| . | SBR | (2008/09) / 2019 | 1.0098 | 0.0285 | 0.3447 | 1.0000 |
| . | BMR | (2000/01) / (2008/09) | 0.9596 | 0.0250 | -1.5828 | 1.0000 |
| . | BMR | (2000/01) / 2019 | 1.0763 | 0.0274 | 2.8913 | 0.2444 |
| . | BMR | (2008/09) / 2019 | 1.1216 | 0.0292 | 4.4167 | 0.0007 |
| . | VD | (2000/01) / (2008/09) | 0.9687 | 0.0233 | -1.3213 | 1.0000 |
| . | VD | (2000/01) / 2019 | 1.0585 | 0.0248 | 2.4252 | 0.9696 |
| . | VD | (2008/09) / 2019 | 1.0927 | 0.0280 | 3.4655 | 0.0341 |
| . | ARA | (2000/01) / (2008/09) | 0.9205 | 0.0273 | -2.7913 | 0.3341 |
| . | ARA | (2000/01) / 2019 | 1.0352 | 0.0295 | 1.2127 | 1.0000 |
| . | ARA | (2008/09) / 2019 | 1.1247 | 0.0311 | 4.2429 | 0.0015 |
| . | SH | (2000/01) / (2008/09) | 0.9845 | 0.0236 | -0.6521 | 1.0000 |
| . | SH | (2000/01) / 2019 | 1.1127 | 0.0265 | 4.4835 | 0.0005 |
| . | SH | (2008/09) / 2019 | 1.1303 | 0.0279 | 4.9556 | 4.95E-05 |
| . | FC | (2000/01) / (2008/09) | 0.9663 | 0.0234 | -1.4153 | 1.0000 |
| . | FC | (2000/01) / 2019 | 1.2624 | 0.0309 | 9.5326 | 2.84E-19 |
| . | FC | (2008/09) / 2019 | 1.3065 | 0.0329 | 10.6124 | 8.47E-24 |

**Table S6** Model results of model II reduced major axis (RMA) regressions comparing snail drilling success (based on a 60±10mm mussel) with mussel shell thickness from the previous decade. Data analyzed were the % of snails within a family that were able to successfully drill at least one mussel during laboratory trials and model estimates from the mussel shell thickness model.

| **Dogwhelk Drilling Time Period** | **Shell Thickness Time Period** | ***r*^2^** | ***P*-value** | **Predictor** | **Estimate** | **95% Confidence Interval of Predictor** |
| --- | --- | --- | --- | --- | --- | --- |
| 2009 | 2000-2001 | 0.532 | 0.100 | Intercept | 649.92 | 303.62,  -2021.33 |
|  |  |  |  | Mussel Thickness | -26397.91 | 91020.17,  -11175.92 |
| 2019 | 2008-2009 | 0.778 | 0.020 | Intercept | 515.62 | 316.59,  1749.86 |
|  |  |  |  | Mussel Thickness | -19451.86 | -71873.06,  -10998.17 |


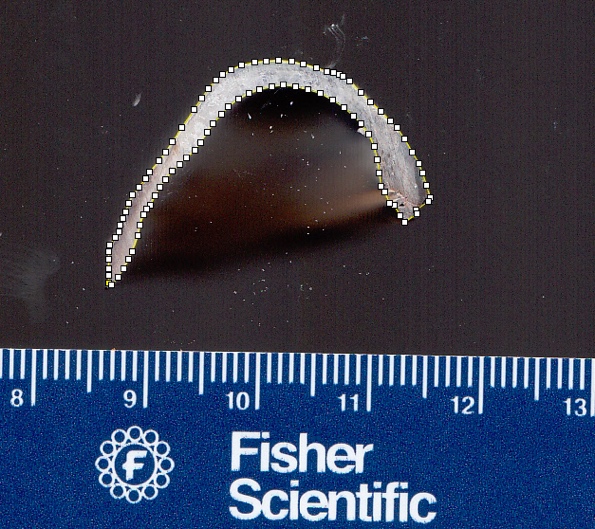

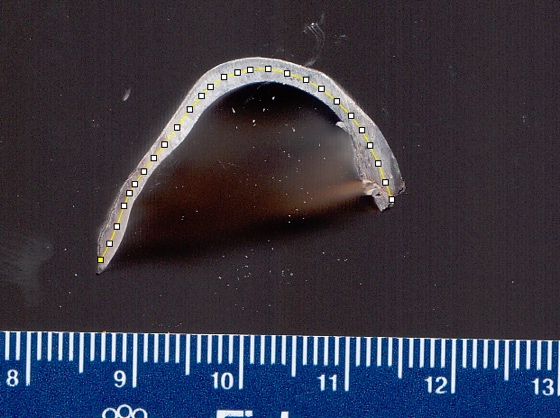

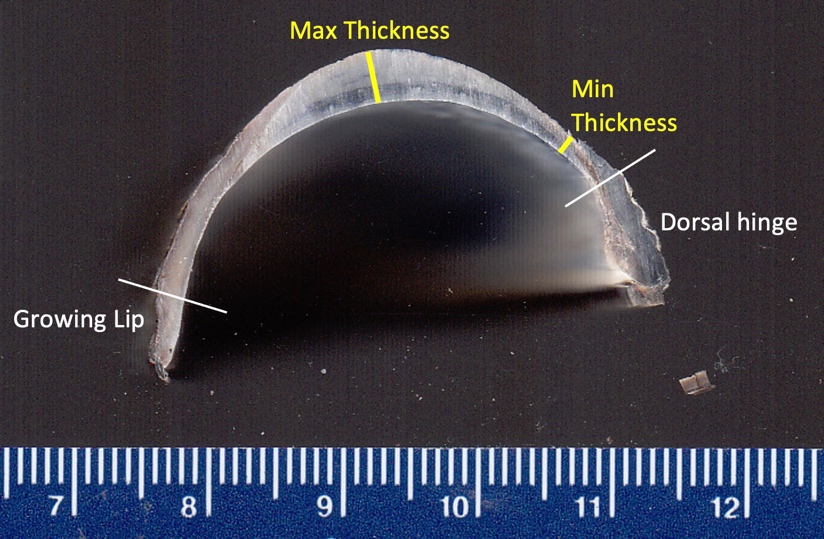


a

b

c

**Fig. S1** Shell thickness measurements of mussels (*Mytilus californianus*). Analyses of mussel shell thickness were conducted using image analysis of cross sections made at 1/3 the length of the mussel (measured from the anterior end). For the 2019 mussels from Soberanes Point and Strawberry Hill, thickness was measured as the area of the polygon around the cross section (a) divided by the length of a segmented line running along the middle of the cross section (b). For the spatial and temporal comparison of mussels from field surveys and for the 8 size classes used in the laboratory experiment, shell thickness was measured as the mean of the maximum and minimum thicknesses, not including the dorsal hinge and growing lip, respectively (c). Scale bars show lengths in mm.

**
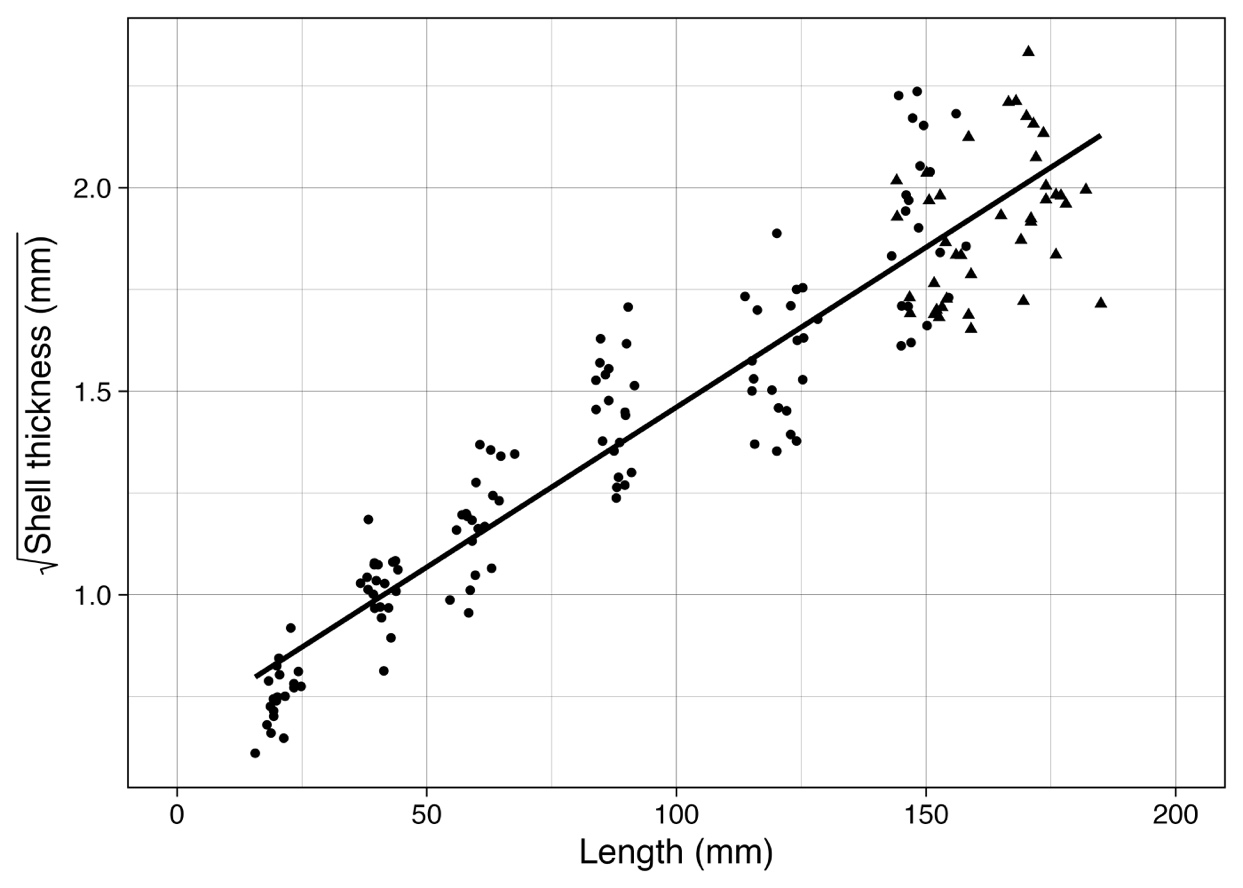
**

**Fig. S2** Variation in shell thickness with mussel length (*Mytilus californianus*). Thickness was measured as the average of the maximum and minimum thickness at 1/3 the mussel’s length disregarding the dorsal hinge and ventral growing lip, respectively. Line is the least squares regression (t_158_ = 34.51, *P* < 0.001, slope = 0.008, *r*^2^ = 0.882). Shell thickness was square root transformed to fit assumptions. Populations are indicated by point shape: circles = Bodega Marine Reserve (BMR); triangles = Strawberry Hill (SH).


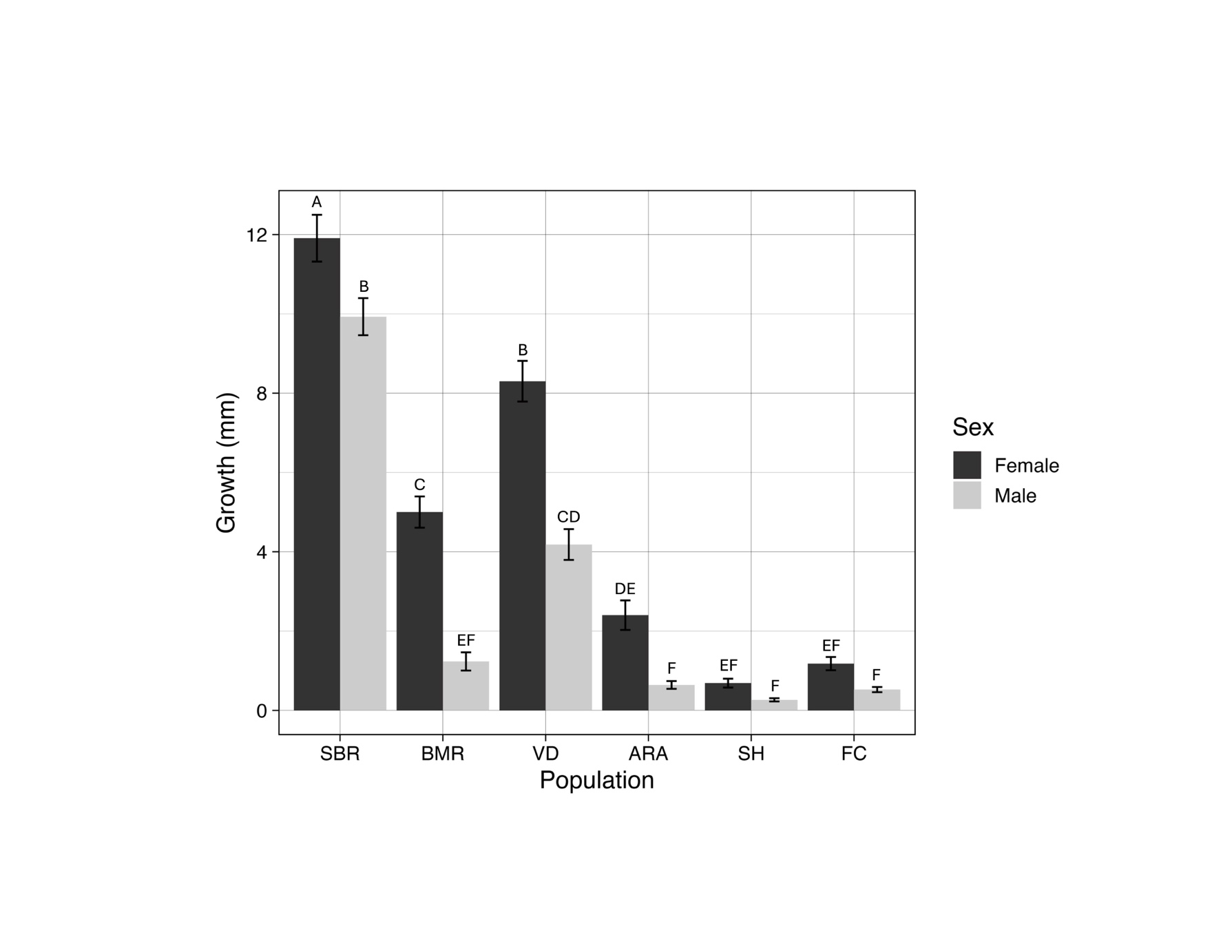


**Fig. S3** Variation in growth (increase in shell length) of the dogwhelks (*Nucella canaliculata*) over the course of the 25-week experiment. Sex of the dogwhelk is depicted with the different colored bars (black = female, grey = male). Shared letters above bars indicate groups that are not significantly different based on the linear mixed effects model (Tukey-test, *P* > 0.05). Sample sizes divided by sex (Female, Male) are as follows: Soberanes Point – 40F, 35M; Bodega Marine Reserve– 49F, 26M; Van Damme – 38F, 35M; Cape Arago – 32F, 45M; Strawberry Hill – 27F, 43M; Fogarty Creek – 29F, 46M.

**
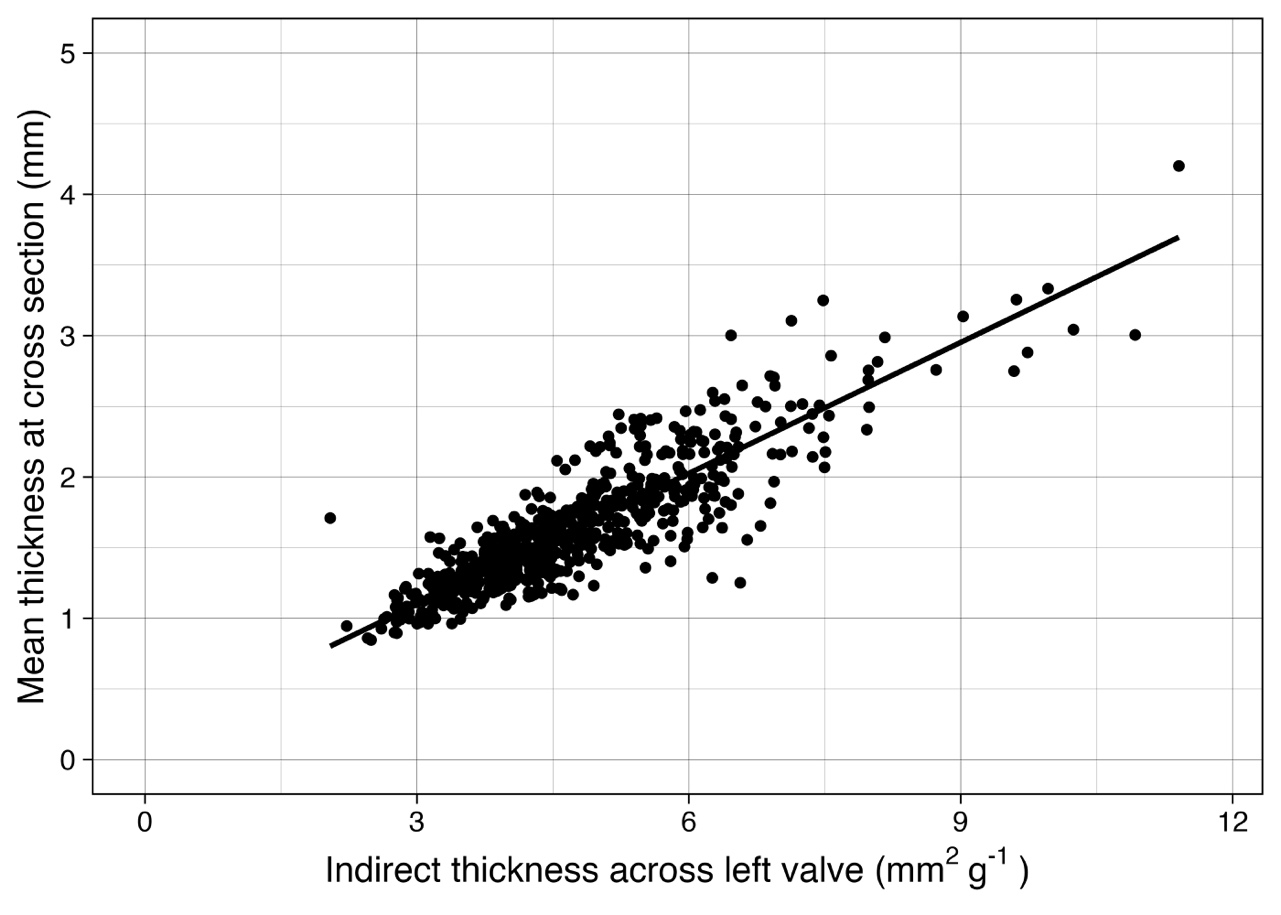
**

**Fig. S4** Mean shell thickness of mussels (*Mytilus californianus*) regressed against indirect mussel shell thickness calculated as the weight of the left valve divided by the surface area of that valve for all 2019 mussel shells. Surface area was estimated as length x (height^2^ + width^2^)^0.5^ x π/2 (following Freeman & Byers 2006). Regression line depicts a model II major axis regression (*P* < 0.001, slope = 0.309, 95% CI of slope: [0.296, 0.323], *r*^2^ = 0.768).

**
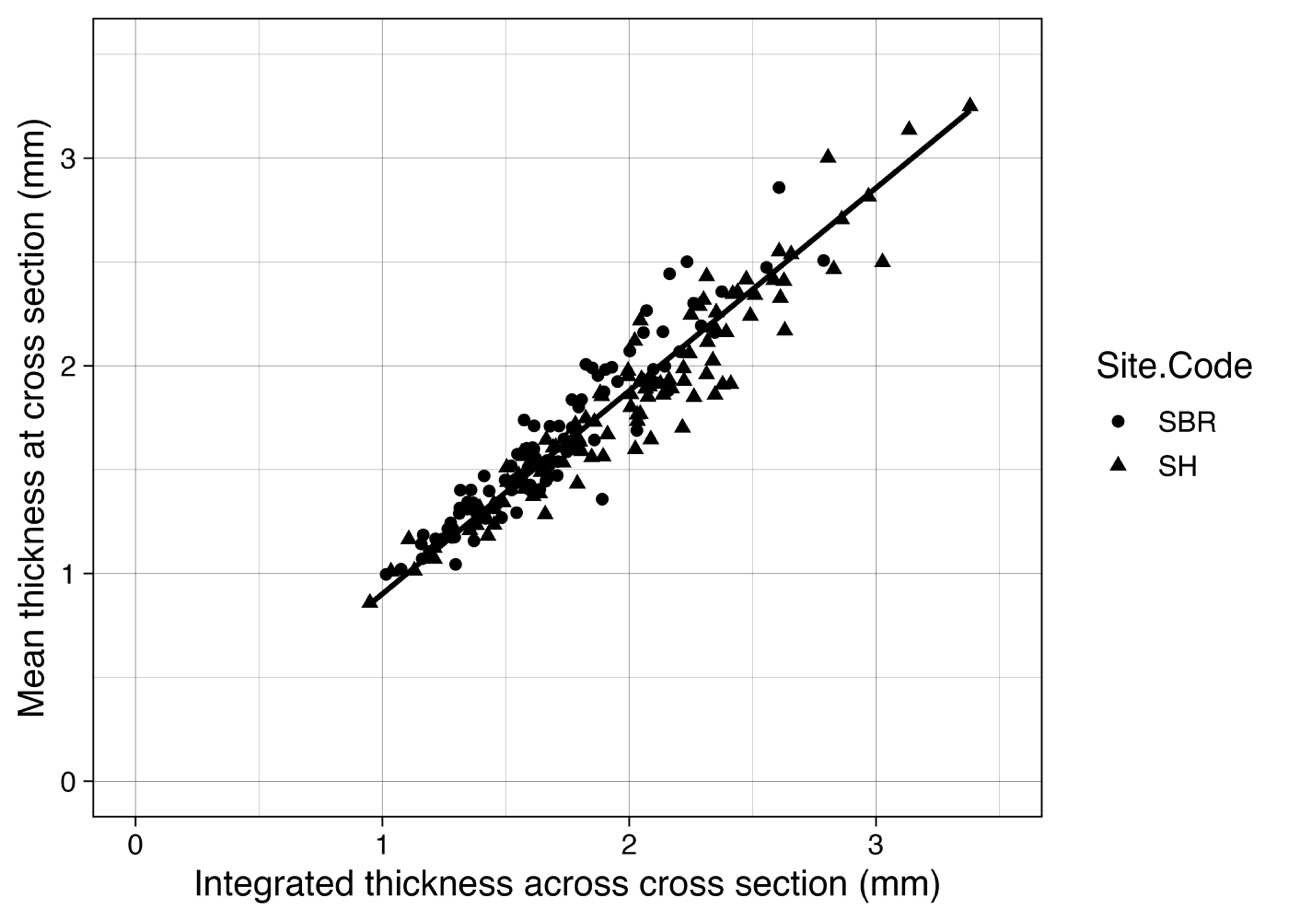
**

**Fig. S5** Mean mussel shell thickness regressed against integrated shell thickness across the entire cross section at 1/3 the length of the mussel. Data are shown for the Soberanes Point (circles) and Strawberry Hill (triangles) populations. Regression line is a model II major axis regression (*P* < 0.001, slope = 0.977, 95% CI of slope: [0.932, 1.024], *r*^2^ = 0.897).

**References**

Freeman AS, Byers JE (2006) Divergent induced responses to an invasive predator in marine mussel populations. Science 313:831-833. https://doi.org/10.1126/science.1125485

Sanford E, Worth DJ (2009) Genetic differences among populations of a marine snail drive geographic variation in predation. Ecology 90:3108-3118. https://doi.org/10.1890/08-2055.1
